# Adaptation of the orthorexia beliefs scale into Turkish: a validity and reliability study

**DOI:** 10.3389/fnut.2026.1877643

**Published:** 2026-07-23

**Authors:** Selen Köksal, İlayda Temizarabacı, Sevde Cantürk, Merve Nur Uçak, Beyza Katırcıoğlu, Gizem Ağır, Perim Fatma Türker, Murat Baş

**Affiliations:** 1 Department of Culinary, Vocational School, Acibadem Mehmet Ali Aydinlar University, İstanbul, Türkiye; 2 Department of Nutrition and Dietetics, Faculty of Health Sciences, Acibadem Mehmet Ali Aydinlar University, İstanbul, Türkiye; 3 Department of Nutrition and Dietetics, Graduate School of Health Sciences, Acibadem Mehmet Ali Aydinlar University, İstanbul, Türkiye

**Keywords:** healthy eating, orthorexia nervosa, orthorexic beliefs, Turkish adaptation, validity and reliability

## Abstract

**Background:**

Orthorexia nervosa is characterized by an excessive preoccupation with healthy eating, rigid dietary rules, and dysfunctional beliefs related to food, health, morality, and control. Despite the growing interest in orthorexia research in Türkiye, instruments directly assessing orthorexic beliefs remain limited. This study aimed to adapt the Orthorexia Beliefs Scale into Turkish and evaluate its psychometric properties in a sample of healthy adults.

**Method:**

This methodological study was conducted between October 2025 and January 2026 using an online snowball sampling strategy. The adaptation process followed a standard forward–backward translation procedure. A pilot study was first performed to assess item clarity and preliminary reliability (*n* = 210). Subsequently, the main study included 1,652 participants, with separate samples allocated for exploratory factor analysis (EFA; *n* = 700) and confirmatory factor analysis (CFA; *n* = 952). Participants completed the OBS, Teruel Orthorexia Scale (TOS), Eating Attitudes Test-26 (EAT-26), Obsessive–Compulsive Inventory-Revised (OCI-R), and Positive and Negative Affect Schedule (PANAS). Internal consistency, construct validity, convergent validity, and criterion validity were evaluated.

**Results:**

The Kaiser–Meyer–Olkin value (0.922) and significant Bartlett’s test of sphericity (χ^2^ = 8,144.004, *p* < 0.001) confirmed sample adequacy for factor analysis. EFA supported a three-factor structure—Overvaluation of Healthy Eating, Need for Control, and Moral Meaning—explaining 53.997% of the total variance. CFA demonstrated good model fit (χ^2^/df = 3.085, RMSEA = 0.047, CFI = 0.981, NFI = 0.972, SRMR = 0.059, GFI = 0.983, AGFI = 0.979). The total scale showed excellent internal consistency (McDonald’s Omega = 0.922). Standardized factor loadings, composite reliability, and average variance extracted values supported convergent validity. OBS total scores were positively associated with orthorexia-related behaviors, obsessive–compulsive symptoms, and affective measures, supporting criterion validity.

**Conclusion:**

The findings indicate that the Turkish version of the Orthorexia Beliefs Scale is a valid, reliable, and culturally appropriate instrument for assessing orthorexic beliefs in the Turkish population and may contribute to future clinical and cross-cultural research on orthorexia nervosa.

## 1 Introduction

Eating behavior represents both a process aimed at meeting biological needs and a complex construct encompassing psychological and sociocultural dimensions. Personal beliefs, past experiences, and the norms of the society in which they live all influence what individuals eat, how they eat, and when they eat (1, 2). In contemporary societies, healthy eating has evolved from a simple health-related behavior to a means of expressing individual identity as well as a social status indicator (3). Although the high value attributed to healthy eating supports public health, it may also, in some individuals, facilitate rigid and excessive eating patterns, thereby creating a basis for psychological issues (4, 5).

In 1997, Steven Bratman first introduced the concept of orthorexia nervosa (ON), which is derived from the Greek words “orthos” (correct) and “orexis” (appetite) (6). Based on his clinical observations, Bratman used the term “orthorexia” to describe individuals who develop obsessive behaviors over time in pursuit of healthy eating. Today, this concept underscores the significance of considering interest in healthy eating across two distinct dimensions: a pathological form known as ON and a nonpathological, more adaptive tendency referred to as healthy orthorexia (HO) (7).

ON is characterized by an excessive focus on the quality, nutritional value, and purity of food; strict dietary rules; and a marked need for control over eating behavior. Individuals with ON highly prioritize the naturalness and cleanliness of food. This condition involves behavioral, cognitive, and emotional components; violations of self-imposed dietary rules may result in feelings of guilt, anxiety, decreased self-esteem, and compensatory behaviors (8–10). By contrast, HO reflects an interest in healthy eating without rigid restrictions or excessive control. However, higher HO levels do not necessarily indicate a better quality diet (7, 11).

A review of measurement tools developed in the field of orthorexia shows that instruments, including the Barcelona Orthorexia Scale, Düsseldorf Orthorexia Scale, and Test of ON, primarily focus on symptom levels and behavioral outcomes (12–14). However, ON should be considered a behavioral eating pattern and a multidimensional construct associated with cognitive, emotional, and clinical psychopathological variables. Therefore, understanding orthorexia necessitates evaluating both symptom severity and the underlying belief systems, eating behavior patterns, obsessive–compulsive traits, and emotional processes. In the literature, the Teruel Orthorexia Scale (TOS) is frequently employed for assessing orthorexia symptoms, the Eating Attitudes Test-26 (EAT-26) for evaluating eating attitudes, and the Obsessive–Compulsive Inventory-Revised (OCI-R) for investigating obsessive–compulsive symptoms. Furthermore, the Positive and Negative Affect Schedule (PANAS) is broadly employed for assessing emotional processes and identifying orthorexia-associated affective patterns (15–18). Among the orthorexia measures adapted into Turkish, the Orthorexia Nervosa Inventory (ONI) and the Düsseldorf Orthorexia Scale (DOS) have demonstrated satisfactory psychometric properties The Turkish ONI retained its original three-factor structure and showed high internal consistency, whereas the Turkish DOS preserved its original unidimensional structure with satisfactory reliability and construct validity (19, 20). However, these instruments mainly focus on orthorexic symptoms and eating behaviors rather than the dysfunctional cognitive beliefs that may underlie these behaviors. Therefore, there remains a need for a valid and reliable instrument capable of directly assessing orthorexic beliefs in the Turkish population. Epidemiological evidence further highlights the clinical relevance of orthorexia, particularly during adolescence. In a large-scale study conducted among 1,784 Turkish high school students aged 14–18 years, the prevalence of ON was reported as 0.5%, and higher orthorexic symptom levels were significantly associated with obsessive–compulsive symptoms, panic symptoms, anxiety, increased body mass index, and authoritarian parenting style. In addition, rural residence, dietary supplement use, and pet ownership were identified as factors associated with higher ON symptom scores. These findings suggest that ON is a multidimensional condition influenced not only by eating-related behaviors but also by cognitive, emotional, familial, and psychosocial factors, emphasizing the importance of comprehensive assessment strategies that extend beyond behavioral symptoms alone (21). Evidence from adult populations in Türkiye further supports the clinical relevance of ON. A cross-sectional study conducted among employees of a private university reported that orthorexic tendencies were relatively common, with younger age and the presence of chronic diseases being associated with a greater likelihood of ON, whereas sex, marital status, body mass index, and working in the healthcare sector were not significantly associated with orthorexic tendencies. These findings indicate that orthorexic behaviors are observed across different adult populations and may be influenced by demographic and health-related characteristics, further highlighting the need for comprehensive assessment tools capable of capturing the cognitive features underlying orthorexic behaviors (22).

Despite these advances, a significant gap exists in the availability of instruments that directly assess the cognitive processes underlying orthorexic behaviors. To overcome this limitation, the Orthorexia Beliefs Scale (OBS), developed by Roncero et al., was designed as a contemporary tool for directly measuring orthorexia-associated cognitive structures. The OBS evaluates orthorexic beliefs across three main dimensions: excessive valuation of healthy eating, moralization of food, and the need for control. The scale comprises 21 items rated on a 4-point Likert scale, with higher scores indicating stronger orthorexic beliefs. The original validation study reported a significant association between the OBS and ON, while showing more limited associations with HO (23). Since its development, the OBS has demonstrated cross-cultural applicability. Notably, a Chinese version of the OBS has been validated, with findings supporting cross-cultural stability of the three-factor structure and similar internal consistency values (24).

Linguistic and cultural adaptation studies are crucial for the appropriate use of measurement tools developed in different cultural contexts. This process involves translation and the reevaluation of the scale’s psychometric properties within the target culture. Although research on orthorexia has been increasing in Türkiye, the number of instruments that directly assess orthorexic beliefs remains limited. In this context, the adaptation of the OBS into Turkish may fill a crucial gap in the field.

This study aimed to adapt the OBS into Turkish by investigating its validity and reliability and evaluate its cultural equivalence and psychometric properties in a sample of healthy adults aged 18–65 years.

## 2 Materials and methods

### 2.1 Participants and study design

This study was conducted from October 2025 to January 2026 to evaluate the Turkish validity and reliability of the OBS. Study data were collected online via Google Forms using a snowball sampling strategy.

This study was conducted in two stages. First, a pilot study was conducted to assess the clarity and comprehensibility of the items, evaluate preliminary reliability, and make necessary design corrections. Subsequently, the main study was performed to investigate the psychometric properties of the scale. Separate samples were collected for the pilot and main studies.

Muthén and Muthén recommend that the sample size should be at least 5–10 times the number of scale items (25). Accordingly, the sample size for the pilot study was 10 times the number of items (*n* = 210). In the pilot study (*n* = 210), participants were equally distributed by sex, with a mean age of 30.6 ± 10.9 years. The pilot sample was considered sufficient to represent the target population in terms of basic sociodemographic characteristics. An a priori power analysis was conducted on the basis of the pilot study findings. The results indicated that the main study required a minimum sample size of 192 participants (effect size *r* = 0.332, α = 0.05, and power = 90%). Following the power analysis, 1,652 participants were recruited for the main study. Separate independent samples were collected for exploratory factor analysis (EFA) and confirmatory factor analysis (CFA). Of these participants, 700 and 952 were included in the EFA and CFA samples, respectively.

Individuals aged ≥ 18 years who voluntarily agreed to participate were included in this study. The Acibadem Mehmet Ali Aydınlar University Medical Research Ethics Committee provided ethical approval (decision number: 2025-13/522). All participants provided written informed consent, and this study was conducted following the Declaration of Helsinki.

A questionnaire was used to evaluate the reliability and validity of the Turkish version of the OBS. The questionnaire encompassed sociodemographic and general characteristics, as well as the OBS, TOS, EAT-26, OCI-R, and PANAS.

### 2.2 Measurements

#### 2.2.1 OBS

The OBS, developed by Roncero et al., comprises 21 items and is used for assessing individuals’ levels of orthorexic beliefs (23). The scale encompasses three subdimensions: Overvaluation of Healthy Eating, Moral Meaning, and Need for Control. Participants responded to each item on a 4-point Likert scale ranging from 0 (strongly disagree) to 3 (strongly agree).

##### 2.2.1.1 Language and cultural adaptation of the OBS

Permission to adapt the OBS was obtained from the original authors. The translation process was conducted using a standard forward–backward translation procedure.

First, two independent bilingual researchers translated the original scale into Turkish. Subsequently, two different bilingual experts back-translated the translated version into English. The original and back-translated versions were compared, and discrepancies were resolved through consensus. To finalize the pretest version, feedback from the original developers was incorporated, ensuring semantic equivalence and linguistic appropriateness for the target population.

#### 2.2.2 TOS

The TOS, developed by Barrada and Roncero and adapted into Turkish by Asarkaya and Arcan, is employed for evaluating problematic (associated with ON) and nonproblematic (HO) attitudes and behaviors associated with healthy eating (7, 26). The instrument is a self-report scale with a 4-point Likert-type response format (0 = strongly disagree, 1 = slightly agree, 2 = moderately agree, and 3 = strongly agree). It comprises 17 items and has a two-factor structure.

#### 2.2.3 EAT-26

The EAT-26, developed by Garner et al., is broadly used for assessing individuals’ eating attitudes (16). In this study, the diet dimension of the scale was utilized, encompassing 26 items. The diet factor includes 13 items, and an example item is “I avoid eating foods with sugar.” Responses are provided on a 6-point Likert scale ranging from 1 (never) to 6 (always). The Cronbach’s alpha coefficient of the scale is 0.86. The Turkish adaptation of the scale, known as the EAT-26 short form, has undergone validity and reliability studies (27).

#### 2.2.4 OCI-R

The OCI-R is a self-report instrument developed by Foa et al. and adapted into Turkish by Yorulmaz et al. to assess obsessive–compulsive disorder symptom-associated distress (17, 28). The scale includes 18 items, each rated on a 5-point Likert scale ranging from 0 (not at all) to 4 (extremely). Total scores range from 0 to 72, with higher scores indicating greater obsessive–compulsive symptom-related distress. Furthermore, the scale evaluates six subdimensions: washing, checking, obsessing, mental neutralizing, ordering, and hoarding.

#### 2.2.5 PANAS

The PANAS, developed by Watson et al. (18), is designed to assess positive and negative affect. It comprises 20 items and includes two factors: positive and negative affect. Items are rated on a 5-point scale (1 = very slightly or not at all, 2 = a little, 3 = moderately, and 4 = quite a bit, 5 = extremely). Gençöz performed the Turkish adaptation, and the internal consistency coefficients were reported as 0.83 and 0.86 for positive and negative affect, respectively (29).

### 2.3 Statistical analysis

Statistical analyses were performed using Statistical Package for the Social Sciences (version 27; IBM Inc., Chicago, IL, USA) and R Project version 3.6.1 (R Core Team, Vienna, Austria).

Categorical variables were presented as frequencies and percentages, and numerical variables were summarized as mean ± standard deviation (SD), median, minimum, and maximum values. The normality of the data was assessed using the Shapiro–Wilk test.

The factor structure of the OBS was evaluated using Exploratory Factor Analysis (EFA). Principal Axis Factoring (PAF) with Promax oblique rotation. was applied to determine the underlying factor structure. Items were assessed based on factor loadings, cross-loadings, and eigenvalues. The Pattern Matrix was examined to evaluate item factor loadings and cross-loadings. Factor loadings greater than 0.30 were considered acceptable, and factors with eigenvalues greater than 1 were retained. Items with a factor loading below 0.30 or with a difference of less than 0.10 between cross-loadings were considered for removal from the scale. Based on these criteria, the final scale structure was determined.

The factor structure of the scale was determined using EFA, and this structure was verified using CFA. Model fit in CFA was evaluated using the following indices: root mean square error of approximation (RMSEA ≤ 0.08), comparative fit index (CFI ≥ 0.90), normed fit index (NFI ≥ 0.90), goodness-of-fit index (GFI ≥ 0.85), adjusted goodness-of-fit index (AGFI ≥ 0.85), and standardized root mean square residual (SRMR < 0.10).

Convergent validity was assessed by investigating standardized factor loadings ( ≥ 0.50), composite reliability (CR ≥ 0.70), and average variance extracted (AVE ≥ 0.50). Criterion validity was evaluated by analyzing the associations between scales using Pearson’s correlation coefficient. Floor and ceiling effects were analyzed to assess the measurement sensitivity and discriminatory capacity of the OBS total and subscale scores. A 15% threshold was used as the standard criterion, and an additional sensitivity analysis was conducted using a 20% threshold to confirm the robustness of the findings.

## 3 Results

### 3.1 EFA

Of the participants included in the EFA (*n* = 700), 19.3 and 80.7% were male and female, respectively. The mean age was 35.66 ± 14.43 years. Most participants were single (56.4%), had an associate or bachelor’s degree (41.3%), and were students (31.3%). The majority reported that their income was equal to their expenses (58.3%), and 20.0% had a chronic disease. The mean body mass index (BMI) was 25.67 ± 4.89 kg/m^2^ (Table 1).

**TABLE 1:** Descriptive statistics of the demographic, health, and BMI characteristics of participants included in the EFA.

	Males (*n* = 135)		Females (*n* = 565)		Total (*n* = 700)	
Characteristic	n	%	n	%	n	%
Age (years) (Mean ± SD)	30.87 ± 13.86		36.80 ± 14.35		35.66 ± 14.43	
Marital Status						
Married	33	24.4	272	48.1	305	43.6
Single	102	75.6	293	51.9	395	56.4
Education Level						
Primary school	6	4.4	51	9.0	57	8.1
Secondary school	9	6.7	33	5.8	42	6.0
High school	51	37.8	192	34.0	243	34.7
Associate/Bachelor’s degree	61	45.2	228	40.4	289	41.3
Master’s/Doctorate	8	5.9	61	10.8	69	9.9
Employment Status						
Public employee	13	9.6	32	5.7	45	6.4
Private sector employee	30	22.2	73	12.9	103	14.7
Worker	10	7.4	34	6.0	44	6.3
Self-employed	15	11.1	32	5.7	47	6.7
Retired	9	6.7	25	4.4	34	4.9
Student	52	38.5	167	29.6	219	31.3
Unemployed	6	4.4	202	35.8	208	29.7
Income Level						
Income < expenses	31	23.0	121	21.4	152	21.7
Income = expenses	58	43.0	350	61.9	408	58.3
Income > expenses	46	34.1	94	16.6	140	20.0
Chronic disease status						
Yes	14	10.4	126	22.3	140	20.0
No	121	89.6	439	77.7	560	80.0
BMI categories						
Underweight	8	5.9	54	9.6	62	8.8
Normal weight	111	82.2	120	21.2	231	33.0
Pre-obese	5	3.7	272	48.1	277	39.6
Obese	11	8.2	119	21.1	130	18.6
BMI (kg/m^2^) (Mean ± SD)	23.54 ± 3.85		26.17 ± 4.97		25.67 ± 4.89	

Multiple responses were allowed. SD, standard deviation; EFA, exploratory factor analysis.

The KMO measure of sampling adequacy for the OBS was 0.922, indicating excellent sampling adequacy. Bartlett’s test of sphericity showed statistical significance (χ^2^ = 8144.004, *p* < 0.001), demonstrating that the data were suitable for EFA. EFA revealed a three-factor structure with factor loadings > 0.30 and eigenvalues > 1, explaining 53.997% of the total variance. No items were removed, as none had factor loadings below 0.30 or problematic cross-loadings (difference < 0.10). The factors were labeled as Overvaluation of Healthy Eating, Need for Control, and Moral Meaning (Table 2). Also, item-total statistics indicated that item-level Omega values ranged from 0.914 to 0.922, suggesting that no items needed to be removed (Table 3).

**TABLE 2:** Factor loadings and structure of the scale based on EFA.

Item	Overvaluation of healthy eating	Need for control	Moral meaning	Communalities
Item 13	0.873			0.739
Item 5	0.863			0.680
Item 6	0.800			0.616
Item 9	0.789			0.631
Item 2	0.742			0.556
Item 4	0.739			0.544
Item 21	0.713			0.532
Item 10	0.687			0.613
Item 1		0.945		0.735
Item 20		0.793		0.604
Item 14		0.773		0.656
Item 17		0.584		0.446
Item 11		0.563		0.424
Item 3		0.519		0.384
Item 19			0.834	0.558
Item 15			0.728	0.575
Item 8			0.713	0.439
Item 18			0.634	0.470
Item 7			0.442	0.385
Item 16			0.432	0.354
Item 12			0.425	0.400
EVO (%)	37.701	9.147	7.149	
Eigenvalue	7.917	1.921	1.501	
ω	0.923	0.861	0.832	

EFA, exploratory factor analysis; EVO, Explained Variance Ratio; ω, McDonald’s Omega Coefficient.

**TABLE 3:** Item-total statistics of the OBS scale based on McDonald’s Omega coefficient.

Item	Scale mean if item deleted	Scale variance if item deleted	Corrected item-total correlation	McDonald’s omega if item deleted
Item1	18,4586	143,156	0,450	0,922
Item2	18,1157	136,941	0,654	0,916
Item3	18,2729	140,502	0,515	0,920
Item4	17,8557	135,351	0,629	0,917
Item5	17,7514	133,477	0,692	0,915
Item6	18,1014	135,940	0,656	0,916
Item7	18,6486	141,278	0,556	0,919
Item8	18,8557	144,719	0,418	0,921
Item9	18,0571	136,283	0,681	0,915
Item10	18,1014	135,476	0,720	0,914
Item11	18,3914	140,396	0,519	0,920
Item12	18,5814	139,889	0,572	0,918
Item13	17,8943	134,229	0,741	0,914
Item14	18,3057	138,979	0,595	0,919
Item15	18,6586	141,086	0,568	0,919
Item16	18,4943	140,270	0,521	0,919
Item17	18,3029	140,841	0,536	0,919
Item18	18,5914	141,630	0,528	0,919
Item19	18,8671	145,143	0,411	0,921
Item20	18,4786	141,689	0,502	0,921
Item21	18,0729	136,345	0,643	0,916

### 3.2 CFA

Of the participants included in the CFA (*n* = 952), 44.4 and 55.6% were male and female, respectively. The mean age was 32.05 ± 12.20 years. Most participants were single (64.0%), had an associate or bachelor’s degree (54.1%), and were students (36.0%). The majority reported that their income was equal to their expenses (51.5%), and 14.9% had a chronic disease. The mean BMI was 24.21 ± 3.99 kg/m^2^ (Table 4).

**TABLE 4:** Descriptive statistics of the demographic, health, and BMI characteristics of participants included in the CFA.

	Males (*n* = 423)		Females (*n* = 529)		Total (*n* = 952)	
Characteristic	*n*	%	*n*	%	*n*	%
Age (years) (Mean ± SD)	36.38 ± 13.39		28.60 ± 9.90		32.05 ± 12.20	
Marital Status						
Married	200	47.3	143	27.0	343	36.0
Single	223	52.7	386	73.0	609	64.0
Education Level						
Primary school	25	5.9	8	1.5	33	3.5
Secondary school	25	5.9	19	3.6	44	4.6
High school	144	34.0	139	26.3	283	29.7
Associate/Bachelor’s degree	198	46.8	317	59.9	515	54.1
Master’s/Doctorate	31	7.3	46	8.7	77	8.1
Employment Status						
Public employee	27	6.4	30	5.7	57	6.0
Private sector employee	121	28.6	97	18.3	218	22.9
Worker	46	10.9	18	3.4	64	6.7
Self-employed	84	19.9	34	6.4	118	12.4
Retired	24	5.7	12	2.3	36	3.8
Student	86	20.3	257	48.6	343	36.0
Unemployed	35	8.3	81	15.3	116	12.2
Income Level						
Income < expenses	82	19.4	105	19.8	187	19.6
Income = expenses	199	47.0	291	55.0	490	51.5
Income > expenses	142	33.6	133	25.1	275	28.9
Chronic Disease Status						
Yes	61	14.4	81	15.3	142	14.9
No	362	85.6	448	84.7	810	85.1
BMI Categories						
Underweight	0	0.0	20	3.8	20	2.1
Normal weight	100	23.6	497	94.0	597	62.7
Pre-obese	245	57.9	12	2.3	257	27.0
Obese	78	18.4	0	0.0	78	8.2
BMI (kg/m^2^) (Mean ± SD)	27.27 ± 3.75		21.76 ± 1.97		24.21 ± 3.99	

CFA, confirmatory factor analysis; SD, standard deviation; BMI, body mass index.

**FIGURE 1:**
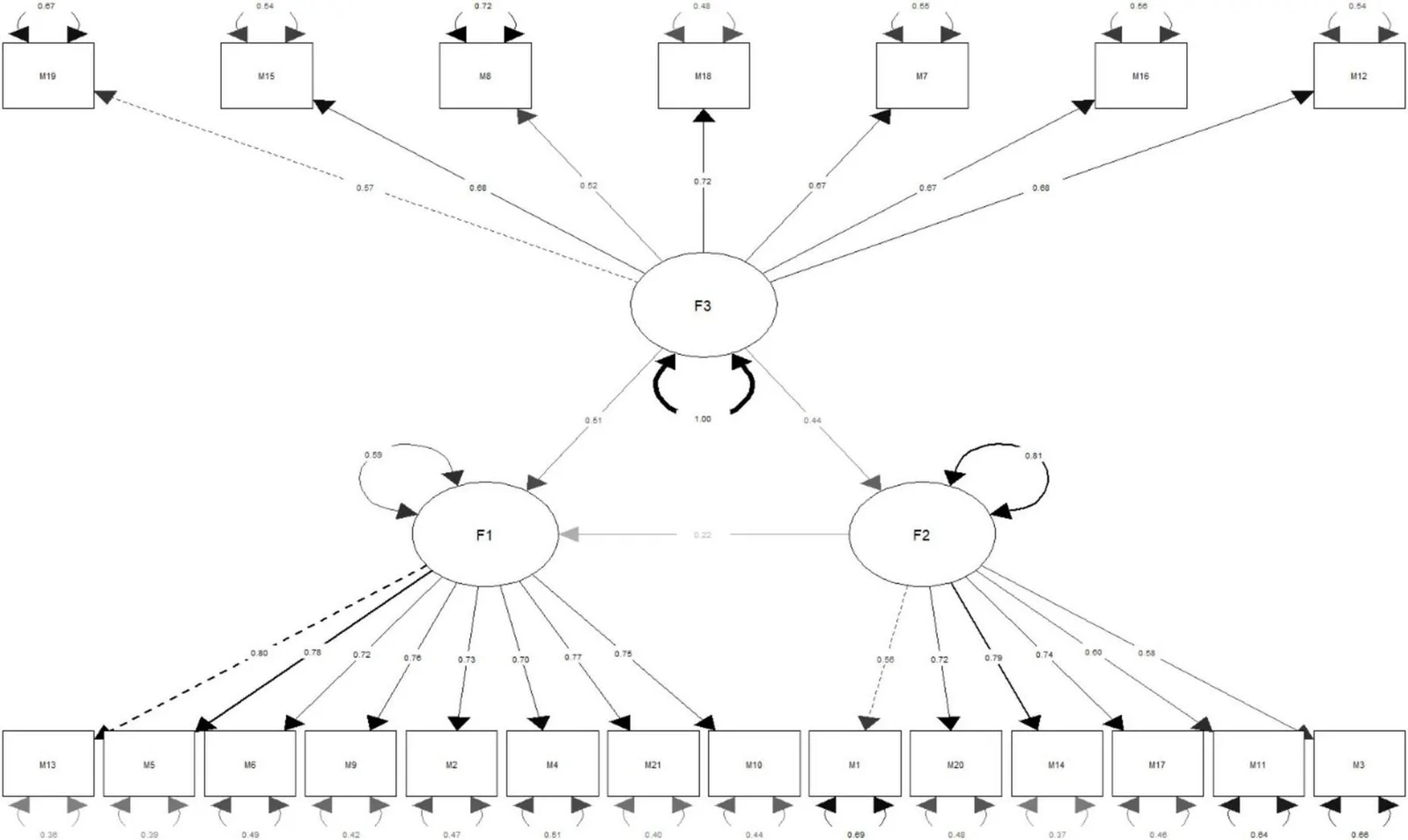
CFA model.

CFA was performed to verify the three-factor structure of the OBS identified in the EFA. The initial model demonstrated acceptable fit indices, and no items had factor loadings below 0.30; therefore, no items were removed (Figure 1).

GFIs indicated a good model fit (χ^2^/df = 3.085, RMSEA = 0.047, NFI = 0.972, CFI = 0.981, SRMR = 0.059, GFI = 0.983, and AGFI = 0.979), with all values within the recommended thresholds (Table 5).

**TABLE 5:** Goodness-of-fit indices of the scale.

Fit index	Threshold values	Analysis results
df	–	186
χ^2^/df	3 ≤ χ^2^/df ≤ 5	3.085
RMSEA	RMSEA ≤ 0.08	0.047
NFI	0.90 ≤ NFI ≤ 1.00	0.972
CFI	0.90 ≤ CFI ≤ 1.00	0.981
SRMR	SRMR < 0.08	0.059
GFI	0.85 ≤ GFI ≤ 1.00	0.983
AGFI	0.85 ≤ AGFI ≤ 1.00	0.979

χ^2^/df, chi-square divided by degree of freedom; RMSEA, root mean square error of approximation; NFI, normed fit index; CFI, comparative fit index; SRMR, standardized root mean square residual; GFI, goodness-of-fit index; AGFI, adjusted goodness-of-fit index.

Regarding floor and ceiling effects, 23 and 29.4% of participants scored within the lower 15 and 20% of the theoretical score range, whereas only 1.1 and 1.3% scored within the upper 15 and 20%, respectively. These findings indicate no ceiling effect but suggest a potential floor effect, which may be attributable to the nonclinical nature of the sample and the relatively low prevalence of strong orthorexic beliefs in the general population (Table 6).

**TABLE 6:** Descriptive statistics of the lowest and highest 15 and 20% OBS total and subscale scores.

		Overvaluation of healthy eating		Need for control		Moral meaning		OBS total	
Cut-off	Group	*n*	%	*n*	%	*n*	%	*n*	%
%15 floor	Below	142	20.3	224	32.0	387	55.3	161	23.0
	Above	558	79.7	476	68.0	313	44.7	539	77.0
%15 ceiling	Above	53	7.6	14	2.0	11	1.6	8	1.1
	Below	647	92.4	686	98.0	689	98.4	692	98.9
%20 floor	Below	167	23.9	257	36.7	448	64.0	206	29.4
	Above	533	76.1	443	63.3	252	36.0	494	70.6
%20 ceiling	Above	70	10.0	18	2.6	11	1.6	9	1.3
	Below	630	90.0	682	97.4	689	98.4	691	98.7

The floor and ceiling effect cut-off values for the subscales of Overvaluation of Healthy Eating, Need for Control, Moral Meaning, and the OBS total score were calculated based on the maximum possible scores for each dimension, respectively 24, 18, 21, and 63 points. For the 15% floor effect, the lower score ranges were defined as 0–3.6, 0–2.7, 0–3.15, and 0–9.45 points, respectively. For the 15% ceiling effect, the upper score ranges were defined as 20.4–24, 15.3–18, 17.85–21, and 53.55–63 points, respectively. For the 20% floor effect, the lower score ranges were defined as 0–4.8, 0–3.6, 0–4.2, and 0–12.6 points, respectively. For the 20% ceiling effect, the upper score ranges were defined as 19.2–24, 14.4–18, 16.8–21, and 50.4–63 points, respectively.

### 3.3 OBS-related results

In this study, the scores of the Overvaluation of Healthy Eating, Need for Control, and Moral Meaning subscales had ranges of 0–24 (*M* = 10.37, SD = 6.37), 0–18 (*M* = 5.15, SD = 3.96), and 0–21 (*M* = 4.58, SD = 4.37), respectively. The total OBS score ranged from 0 to 62 (*M* = 20.10, SD = 11.69) (Table 7).

**TABLE 7:** Descriptive statistics of the subscale and total scores of the OBS.

Subscales	n	M*ean*±S*D*	Median (min–max)
Overvaluation of healthy eating	952	10.37 ± 6.37	10 (0–24)
Need for control	952	5.15 ± 3.96	5 (0–18)
Moral meaning	952	4.58 ± 4.37	3.5 (0–21)
OBS total	952	20.10 ± 11.69	20 (0–62)

SD, standard deviation; OBS, Orthorexia Beliefs Scale.

### 3.4 Associations among the OBS, TOS, PANAS, and OCI-R

OBS total scores showed significant positive correlations with TOS Healthy Orthorexia, TOS Orthorexia Nervosa, and TOS total scores (all *p* < 0.001). OBS total scores were moderately correlated with OCI-R total scores and weakly correlated with PANAS positive and negative affect scores (*p* < 0.001) (Table 8). These findings support the criterion validity of the scale.

**TABLE 8:** Correlation coefficients between OBS subscales and total scores, TOS, PANAS, and OCI-R scores.

Variable		1	2	3	4	5	6	7	8	9	10
1. Overvaluation of healthy eating	*r*	1	1	1	1	1	1	1	1	1	1
	*p*	–									
2. Need for control	*R*	0.388									
	*P*	< 0.001***	–								
3. Moral meaning	*r*	0.525	0.365								
	*p*	< 0.001***	<0.001***	–							
4. OBS total	*r*	0.872	0.686	0.783							
	P	< 0.001***	<0.001***	< 0.001***	–						
5. TOS: healthy orthorexia	*r*	0.631	0.313	0.610	0.678						
	*p*	< 0.001***	<0.001***	< 0.001***	<0.001***	–					
6. TOS: orthorexia nervosa	*r*	0.616	0.299	0.606	0.664	0.877					
	*p*	< 0.001***	<0.001***	< 0.001***	<0.001***	< 0.001***	–				
7. TOS total	*r*	0.632	0.322	0.639	0.692	0.975	0.956				
	*p*	< 0.001***	<0.001***	< 0.001***	<0.001***	< 0.001***	<0.001***	–			
8. PANAS: positive affect	*r*	0.422	0.188	0.225	0.378	0.356	0.330	0.351			
	*p*	< 0.001***	<0.001***	< 0.001***	<0.001***	< 0.001***	<0.001***	< 0.001***	–		
9. PANAS: negative affect	*r*	0.054	0.275	0.222	0.206	0.163	0.178	0.185	0.174		
	*p*	0.094	< 0.001***	<0.001***	< 0.001***	<0.001***	< 0.001***	<0.001***	< 0.001***	–	
10. OCI-R total	*r*	0.372	0.411	0.294	0.452	0.366	0.378	0.383	0.337	0.389	
	*p*	< 0.001***	<0.001***	< 0.001***	<0.001***	< 0.001***	<0.001***	< 0.001***	<0.001***	< 0.001***	–

OBS, Orthorexia Beliefs Scale; TOS, Teruel Orthorexia Scale; PANAS, Positive and Negative Affect Schedule; OCI-R, Obsessive–Compulsive Inventory-Revised. r, Pearson correlation coefficient, p, significance level.

***p < 0.001.

## 4 Discussion

ON is an eating-related condition characterized by an obsessive preoccupation with “healthy” or “pure” foods, resulting in rigid self-imposed dietary rules and severe distress when these rules are not met (30). ON is gaining attention because it can lead to nutritional deficiencies, weight loss, anxiety, depression, and reduced quality of life (31, 32). This study evaluated the validity and reliability of the OBS, which was developed to enhance understanding of the structure of healthy eating-related dysfunctional beliefs and their association with orthorexia, in the Turkish population. Orthorexia represents a behavioral eating disorder and a multidimensional cognitive construct focused on the concepts of health, control, and morality (23). As tools for measuring orthorexia-related cognitive beliefs are limited in Türkiye, this study aimed to significantly contribute to the literature.

First, the dataset was evaluated for missing data, outliers, and the normal distribution assumption. Subsequently, the suitability of the data for factor analysis was assessed using the KMO coefficient and Bartlett test (33). A 0.922 KMO value indicates excellent sample adequacy as it is well above the recommended threshold of 0.70 (34). The significant findings of the Bartlett test (*p* < 0.001) confirmed that the dataset was suitable for factor analysis. The analysis results revealed that no items required removal, highlighting the scale’s homogeneous structure and robust conceptual coherence. All items were retained, as each exceeded the minimum acceptable factor loading threshold ( ≥ 0.30) and demonstrated no difficult cross-loadings, thereby preserving the conceptual and structural integrity of the original scale.

Following this, factor loadings were evaluated for the EFA, and it was observed that all items had high loading values ( > 0.5) on the factors to which they belonged. The literature indicates that the minimum acceptable value for factor loadings is 0.30 (35). EFA revealed a stable three-factor structure that conceptually aligns with the original 21-item OBS structure developed by Roncero (23). These factors, including Overvaluation of Healthy Eating, Need for Control, and Moral Meaning, explain 53.997% of the total variance. They account for 37.701, 9.147, and 7.149% of the total variance, respectively. This three-factor structure shows that orthorexia is not only about health but also encompasses control and moral value-associated psychological patterns. The obtained three-factor structure aligns with the original scale and other cultural adaptation studies (23, 24).

Chinese version supported cross-cultural stability of the scale (24). In the present study, the 21-item OBS showed high reliability, with a McDonald’s Omega coefficient of 0.922 indicates excellent internal consistency, exceeding the recommended threshold ( > 0.80) for high reliability (36). CFA results, including χ^2^/df = 3.085, RMSEA = 0.047, and CFI = 0.981, fully align with the good-fit criteria recommended in the literature (37). These results suggest that the proposed three-factor model demonstrates satisfactory statistical fit and theoretical coherence. To evaluate the criterion validity of the scale, correlation analyses were conducted following the method used in the original OBS study. OBS scores exhibited significant positive associations with measures of TOS, OCI-R, and disordered eating, supporting the validity of the construct. Notably, the magnitude and direction of these correlation coefficients were largely consistent with those reported by Roncero et al. (23), confirming a comparable pattern of associations across different cultural contexts and reinforcing the theoretical consistency of the OBS-Turkish version.

These psychometric findings are also consistent with those reported for other validated Turkish orthorexia measures. The Turkish adaptation of the Orthorexia Nervosa Inventory (ONI) similarly retained its original three-factor structure (behaviors, impairments, and emotions) and demonstrated good internal consistency (Cronbach’s α = 0.91) together with acceptable model fit indices (CFI = 0.94, RMSEA = 0.08) (19). Likewise, the Turkish version of the Düsseldorf Orthorexia Scale (DOS) preserved its original unidimensional structure and showed satisfactory reliability (Cronbach’s α = 0.85; ω = 0.85) and construct validity. Compared with these instruments, the OBS also demonstrated strong psychometric performance, with excellent model fit (CFI = 0.981, RMSEA = 0.047) while preserving the original three-factor structure without requiring item removal (20).

While the moderate-to-strong positive correlations between the OBS and orthorexia-related measures (e.g., the TOS) provide evidence for criterion validity, they also highlight the importance of distinguishing orthorexic beliefs from orthorexic symptoms. Unlike existing instruments (e.g., ONI and DOS) that primarily assess behavioral manifestations and symptom severity, the OBS was developed to evaluate the underlying cognitive beliefs related to healthy eating, moral meaning, and the need for control. Therefore, the observed correlations should be interpreted as evidence of conceptual relatedness rather than construct redundancy. In other words, stronger orthorexic beliefs are expected to be associated with greater orthorexic symptomatology while still representing a distinct cognitive construct that complements existing symptom- and behavior-oriented measures. Similarly, Turkish validation studies of the ONI and DOS conducted in adolescent populations have also demonstrated satisfactory psychometric properties, suggesting that orthorexia assessment tools can be reliably applied across different developmental stages (38, 39). Future studies should investigate whether the OBS demonstrates comparable psychometric performance in adolescent samples.

The finding that the Overvaluation of Healthy Eating factor accounts for the largest proportion of variance (37.701%) further supports this view, as this dimension was also the strongest correlate of orthorexic beliefs in the original study (23). Consistent with the broader literature (40), our findings suggest that the cognitive Overvaluation of healthy eating forms a core component of orthorexia in the Turkish cultural context. This pattern may be partly explained by recent evidence suggesting that social media impacts nutritional awareness, directly or indirectly influencing dietary behavior by amplifying attention to healthy eating trends (31, 41).

Furthermore, the emergence of Need for Control as a distinct factor emphasizes that orthorexia is not solely associated with food choices but is also closely linked to broader psychological mechanisms, including the regulation of uncertainty and the need to maintain control over one’s life. In the original OBS study, Need for Control was also identified as a crucial ON determinant (23). Previous studies have similarly underscored that control-related concerns and loss of control-associated anxiety constitute core components in ON psychopathology (42, 43). Moreover, recent evidence suggests that perfectionism plays a key role in reinforcing rigid control behaviors observed in orthorexia (44, 45). Supporting these findings, Kater et al. reported that anxiety levels were positively associated with ON in a community-based Turkish sample. This finding may be linked to the belief among participants with a higher ON predisposition that adhering to healthy eating habits can reduce stress (30, 46).

Collectively, these findings indicate that control-oriented cognitive processes, rather than food-related concerns alone, can play a crucial role in the development and maintenance of orthorexic tendencies. It is important to interpret these robust factor structures and high internal consistency within the context of our sampling strategy. The use of an online snowball sampling approach resulted in a study sample predominantly composed of younger, highly educated individuals and students. Such demographic homogeneity may have contributed to more consistent interpretations of the scale items, potentially facilitating the observed factor structure and high internal consistency. Likewise, the prominence of the Overvaluation of Healthy Eating and Need for Control dimensions may partly reflect the characteristics of the study sample rather than those of the broader and more heterogeneous Turkish population. Therefore, although the psychometric findings support the validity and reliability of the OBS-Turkish version, their generalizability should be interpreted with appropriate caution.

The Moral Meaning subscale captures the tendency to imbue dietary choices with ethical weight and social status. Orthorexia is considered a socioculturally shaped phenomenon wherein eating behaviors are employed for maintaining a sense of control and constructing a moralized sense of personal identity based on healthy living (47). These findings further support the conceptual relevance of the Moral Meaning dimension and highlight the importance of considering value-based cognitive processes when evaluating orthorexic beliefs.

### 4.1 Strengths and limitations

To our knowledge, this study represents the first Turkish adaptation and validation of the OBS, thereby expanding the restricted methodological resources available for orthorexia research in Türkiye by introducing a culturally appropriate and psychometrically robust measurement tool. The OBS-Turkish version demonstrates satisfactory reliability and validity, facilitating the assessment of key cognitive dimensions, including the Overvaluation of Healthy Eating, Moral Meaning, and Need for Control.

This study had several limitations that should be considered when interpreting the findings. First, the cross-sectional design precluded causal inferences regarding the associations between orthorectic beliefs and related psychological constructs, as well as the temporal stability of these associations. Second, data were collected through online platforms using a snowball sampling strategy, which may have introduced selection bias and limited the representativeness of the sample. Individuals who are more interested in healthy eating, nutrition, or psychological topics and those who are more active on social media may have been more likely to participate. Consequently, the observed levels of orthorexic beliefs and the relationships between variables may not fully reflect those of the general Turkish population. Furthermore, the sample was heavily skewed toward females and younger individuals, as another limitation of this study. This imbalance may considerably limit the generalizability of the findings, particularly to male participants and older adults. Additionally, the marked difference in sex distribution between the EFA and CFA subsamples potentially impacted the comparability of the factor structures, although this difference arose from random sampling rather than intentional stratification. Third, the reliance on self-report measures may have introduced response biases, including social desirability and subjective reporting tendencies. Moreover, although the sample size was sufficient for psychometric analyses, the absence of clinically diagnosed participants restricts the ability to evaluate the discriminative power of the scale between clinical and nonclinical populations. Another limitation of the present study is the absence of test-retest reliability assessment. While internal consistency was established, the temporal stability of the OBS-Turkish version was not evaluated. Future research should incorporate a test-retest design with an interval of 2–4 weeks to provide evidence for the stability of orthorexic belief measurements over time. Finally, although criterion validity was supported through correlation analyses, future research would benefit from incorporating objective or behavioral measures to further strengthen the external validity of the scale. To better assess the predictive validity and diagnostic utility of orthorectic beliefs over time, future studies employing longitudinal designs and more diverse, including clinical, samples are recommended.

In conclusion, this study is the first to validate the Turkish version of the OBS, confirming that it is a psychometrically robust and culturally appropriate instrument for assessing the cognitive dimensions of ON in this Turkish sample. EFA and CFA supported a stable three-factor structure (Overvaluation of Healthy Eating, Need for Control, and Moral Meaning) consistent with the original theoretical model, demonstrating robust structural coherence. The significant associations with orthorexia-related measures, obsessive–compulsive symptoms, and affective variables further support the criterion validity of the scale. Overall, the OBS provides a reliable tool for assessing orthorectic beliefs in Turkish community samples and may facilitate future research and clinical practice, although additional validation in more representative and clinical populations is needed. In addition to its research applications, the OBS-Turkish version may also support clinical practice by providing a standardized approach to assessing orthorectic beliefs. Given that orthorexia nervosa has not yet been formally recognized as a distinct diagnostic disorder, healthcare professionals—including psychiatrists, psychologists, and dietitians—may differ in their recognition, conceptualization, and management of orthorexic presentations. A valid instrument targeting the cognitive dimensions of orthorexia may facilitate more consistent assessment, enhance interdisciplinary communication, and contribute to the early identification of individuals with clinically significant orthorectic beliefs. Future research should explore the diagnostic utility of the OBS-Turkish version in clinical cohorts and investigate how these cognitive beliefs evolve across various life stages in diverse cultural settings.

## Data Availability

The raw data supporting the conclusions of this article will be made available by the authors, without undue reservation.
