# Correction: Adaptation of the orthorexia beliefs scale into Turkish: a validity and reliability study

**DOI:** 10.3389/fnut.2026.1947568

**Published:** 2026-08-07

**Authors:** Selen Köksal, İlayda Temizarabacı, Sevde Cantürk, Merve Nur Uçak, Beyza Katırcıoğlu, Gizem Ağır, Perim Fatma Türker, Murat Baş

**Affiliations:** 1 Department of Culinary, Vocational School, Acibadem Mehmet Ali Aydinlar University, Istanbul, Türkiye; 2 Department of Nutrition and Dietetics, Faculty of Health Sciences, Acibadem Mehmet Ali Aydinlar University, Istanbul, Türkiye; 3 Department of Nutrition and Dietetics, Graduate School of Health Sciences, Acibadem Mehmet Ali Aydinlar University, Istanbul, Türkiye

**Keywords:** healthy eating, orthorexia nervosa, orthorexic beliefs, Turkish adaptation, validity and reliability

**Affiliation** ^3^Department of Nutrition and Dietetics, Graduate School of Health Sciences, Acibadem Mehmet Ali Aydinlar University, Istanbul, Türkiye was omitted from the paper. This affiliation has now been added for authors “İlayda Temizarabacı, Sevde Cantürk, Merve Nur Uçak, Beyza Katırcıoğlu, Gizem Ağır, Perim Fatma Türker, and Murat Baş.”

The original version of this article has been updated.

